# Ventral Midbrain NMDA Receptor Blockade: From Enhanced Reward and Dopamine Inactivation

**DOI:** 10.3389/fnbeh.2016.00161

**Published:** 2016-08-26

**Authors:** Giovanni Hernandez, Marie-Pierre Cossette, Peter Shizgal, Pierre-Paul Rompré

**Affiliations:** ^1^Département de Neurosciences, Université de MontréalMontréal, QC, Canada; ^2^FRQ-S Research Group in Behavioral Neurobiology, Department of Psychology, Concordia UniversityMontréal, QC, Canada

**Keywords:** dopamine, glutamate, NMDA, reward, *in vivo* voltammetry

## Abstract

Glutamate stimulates ventral midbrain (VM) N-Methyl-D-Aspartate receptors (NMDAR) to initiate dopamine (DA) burst firing activity, a mode of discharge associated with enhanced DA release and reward. Blockade of VM NMDAR, however, enhances brain stimulation reward (BSR), the results can be explained by a reduction in the inhibitory drive on DA neurons that is also under the control of glutamate. In this study, we used fast-scan cyclic voltammetry (FSCV) in anesthetized animals to determine whether this enhancement is associated with a change in phasic DA release in the nucleus accumbens. Rats were implanted with a stimulation electrode in the dorsal-raphe (DR) and bilateral cannulae above the VM and trained to self-administer trains of electrical stimulation. The curve-shift method was used to evaluate the effect of a single dose (0.825 nmol/0.5 μl/side) of the NMDAR antagonist, (2R,4S)-4-(3-Phosphopropyl)-2-piperidinecarboxylic acid (PPPA), on reward. These animals were then anesthetized and DA release was measured during delivery of electrical stimulation before and after VM microinjection of the vehicle followed by PPPA. As expected, phasic DA release and operant responding depended similarly on the frequency of rewarding electrical stimulation. As anticipated, PPPA produced a significant reward enhancement. Unexpectedly, PPPA produced a decrease in the magnitude of DA transients at all tested frequencies. To test whether this decrease resulted from excessive activation of DA neurons, we injected apomorphine 20 min after PPPA microinjection. At a dose (100 μg s.c.) sufficient to reduce DA firing under control conditions, apomorphine restored electrical stimulation-induced DA transients. These findings show that combined electrical stimulation and VM NMDARs blockade induce DA inactivation, an effect that indirectly demonstrates that VM NMDARs blockade enhances reward by potentiating stimulation-induced excitation in the mesoaccumbens DA pathway.

## Introduction

Glutamate, the major excitatory neurotransmitter in the brain, plays a major role in behavioral, cognitive and motivational functions. Within the ventral midbrain (VM), glutamatergic afferents potently modulate reward-relevant circuitry by controlling dopamine (DA) excitability via two opposing mechanisms. Through its action on GABAergic afferents and GABAergic interneurons, glutamate maintains a strong inhibitory drive on DA neurons (Grace et al., 2007) so that a majority of them are unresponsive to excitatory inputs (Grace and Bunney, 1984). In contrast, through its direct action on DA neurons, glutamate switches DA neural activity from a slow, irregular, firing pattern to a phasic burst-firing mode that is associated with enhanced DA release (Grace and Bunney, 1984; Charara et al., 1996; Geisler et al., 2007; Omelchenko et al., 2009). Moreover, this mode of neural activity is associated with the acquisition of appetitive and aversive tasks (Zweifel et al., 2009). It has also been proposed that DA burst firing encodes reward prediction errors (Montague et al., 1996) and conveys motivationally relevant signals to anterior forebrain regions that control executive functions (Overton and Clark, 1997).

Excitation and inhibition of DA neurons may entail multiple glutamate receptor subtypes with different sensitivity to agonists and antagonists and the expression on different inputs to VM DA neurons. Empirical evidence for such an arrangement comes from experiments in which systemic or local injection of either N-methyl-D-aspartate receptor (NMDAR) agonists or antagonists increases DA burst firing (French et al., 1993), terminal DA release (Westerink et al., 1996; Mathé et al., 1998; Kretschmer, 1999), and forward locomotion (Kretschmer, 1999; Cornish et al., 2001). Furthermore, VM microinjections of short inhibitory RNA that reduces the number of NMDAR on VM neurons results in an attenuation of reward induced by dorsal-raphe (DR) electrical stimulation, while VM microinjection of (2R,4S)-4-(3-Phosphopropyl)-2-piperidinecarboxylic acid (PPPA) and 2-carboxypiperazin-4-propyl-1-phosphonic acid ((R)-CPP), NMDAR antagonists that display a high affinity for receptor composed of the GluN2A subunits, produces the opposite effect (Bergeron and Rompré, 2013; Hernandez et al., 2015). The most likely mechanism for the reward-attenuating effect is a reduction in the NMDARs that control DA burst firing, whereas a likely mechanism for the reward-enhancing effect is a blockade of a different subtype of NMDARs that maintain the inhibitory drive on DA neurons. Given the large body of evidence supporting a role of VM mesoaccumbens DA neurons in reward (Wise and Rompre, 1989; Lak et al., 2014; Eshel et al., 2016) and the more recent evidence that activation of VM glutamatergic inputs from the DR induces reward and enhances extracellular DA in the nucleus accumbens (Qi et al., 2014), we used fast-scan cyclic voltammetry (FSCV) to determine whether the enhancement of DR reward by VM NMDAR blockade is associated with a change in phasic DA release in the nucleus accumbens shell (NAcS).

## Materials and Methods

### Subjects and Surgery

**Figure 1:**
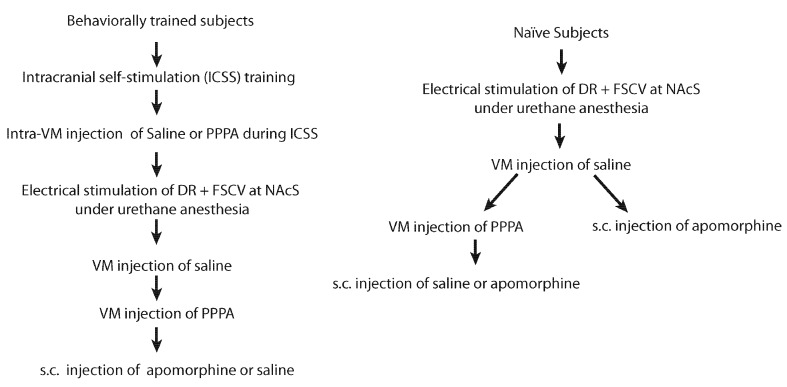
**Experimental sequence for behaviorally trained and naïve subjects**.

Sixteen (16) male Long-Evans rats (Charles River, St-Constant, QC, Canada) weighing between 350–400 g at the time of the surgery were used. Rats were individually housed in a temperature- and humidity- controlled room with a 12-h light-dark cycle (lights on at 06:00 h) and *ad libitum* access to food and water. After a minimum 7-day period of acclimation to the housing environment rats were anesthetized with isoflurane and stereotaxically implanted according to Paxinos and Watson (2007) coordinates with 26-gauge guided cannulae (HRS Scientific, Montreal, Canada) aimed bilaterally at the VM (−5.5 mm AP, ±3.2 mm ML at a 18° angle, −6.5 mm DV from the skull surface) and a stainless steel monopolar electrode aimed at the DR (−7.6 mm AP, 0 mm ML, −6.6 mm DV from the skull surface). Detailed surgical procedures can be found in Bergeron and Rompré (2013). Of these 16 rats, nine rats were trained to nose-poke to deliver electrical pulses to the DR; whereas the other seven were used only in the electrochemistry experiment. For the behaviorally trained rats, one failed to self-stimulate and in three rats we were unable to measure DA. For the non-behaviorally trained rats we were unable to measure DA in one rat. For an schematic of the experimental sequence see Figure 1. All procedures were approved by the Animal Care and Use Committee of the Université de Montréal and Concordia University in accordance with the guidelines of the Canadian Council on Animal Care.

### Drugs

PPPA (Tocris, Ellisville, MI, USA) was dissolved in sterile 0.9% saline and stored frozen in 40–50 μl aliquots. Drug solutions were thawed just before testing and used only once. PPPA was injected into the VM at a dose of 0.825 nmol/0.5 μl/side. Urethane (ethyl carbamate; Sigma, St. Louis, MO, USA) was dissolved in sterile 0.9% saline; it was injected intraperitoneally (i.p.) at a dose of 1.5 g/kg. Apomorphine (Sigma, St. Louis, MO, USA) was dissolved in sterile 0.9% saline; it was injected subcutaneously (s.c.) at a total dosage of 100 μg /kg. Drug doses are expressed as salts.

### Self-Stimulation Training

Eight rats were shaped to nose poke, under a fixed ratio 1 (FR1), for a 0.4-s train of cathodal, rectangular, constant-current pulses, 0.1 ms in duration, delivered at a frequency of 98 Hz. Once the rat nose poked consistently at current intensities between 125 and 400 μA, a rate vs. pulse-frequency curve was obtained by varying the stimulation frequency across trials over a range that drove the number of rewards earned from maximal to minimal levels. A detailed shaping and training procedure can be found in Hernandez et al. (2015).

At least three behavioral tests were carried out before the FSCV experiments. A first saline test was performed to habituate the animals to the microinjection procedure. The detailed bilateral injections procedure can be found in Hernandez et al. (2015). Immediately after the microinjection, rats were allowed to self-stimulate for an hour. Results from this test were not included in the analysis. Baseline data were collected 1 week after this test. The rate vs. pulse-frequency data were fitted using the following sigmoid function

$$y\;=\;Min+\frac{\left(Max-Min\right)}{1+10^{\left(x50-x\right)*p}}$$

where *Min* is the lower asymptote, *Max* is the upper asymptote, *x50* is the position parameter denoting the frequency at which the slope of the curve is maximal, and* p* determines the steepness of the sigmoid curve. The resulting fit was used to derive an index of reward threshold, defined as the pulse-frequency sustaining a half-maximal rate of responding (M50). Self-stimulation behavior was considered stable when the M50 values varied less than 0.1 log unit for three consecutive days. Once stable performance was obtained, we evaluated, on separate days, the effect of bilateral VM microinjections of a single dose (0.825 nmol/0.5 μl/side) of PPPA and an equal volume of saline (counterbalance order).

### FSCV

After at least 4 days after completion of the last behavioral test, rats were anesthetized, and phasic DA release was measured during delivery of DR stimulation, both before and after bilateral VM microinjection of saline and PPPA. Rats were anesthetized with urethane and placed in the stereotaxic apparatus. Holes were drilled for the placement of the carbon-fiber electrode, sintered Ag/AgCl reference electrode (*In Vivo* Metrics, Healdsburg, CA, USA) and the anode electrode. For naïve rats (not behaviorally tested; *n* = 7), a DR stimulation electrode and VM cannulae were also implanted using the stereotaxic coordinates mentioned above. The carbon fiber electrode was built by encasing a carbon fiber (Thorne, Amoco Corporation, Greenville, SC, USA) in a single barrel borosilicate glass capillary (ID = 0.40 mm OD = 0.60 mm; A-M System Carlsborg, WA, USA). The seal between the carbon fiber and the glass was produced by heating the glass capillary with a pipette puller (PUL-1, WPI, Sarasota, FL, USA). A wire covered with silver paint (GC Electronics, Rockford, IL, USA) was inserted in the capillary to make contact with the carbon fiber and secured with shrink tubing coated with epoxy. The carbon fiber electrodes had an exposed tip length of 150–200 μm exposed tip length and 7 μm diameter. The carbon-fiber electrode (working electrode) was aimed at the NAcS (+1.7 mm AP, +1 mm ML, −7.0 mm DV). FSCV was computer- controlled as described previously (Heien et al., 2003). In brief, an 8.5 ms triangular input waveform (initial ramp, −0.4 to 1.3 V, 400 V/s; Heien et al., 2003) was applied to the working electrode at 10 Hz. The potential was held at −0.4 V between each scan to promote cation absorption at the surface of the FSCV electrode. A computer using software written in LABVIEW (National Instruments) and a multifunction data-acquisition board (PCI-6052E, National Instruments) controlled the waveform parameters and digitalized the recorded data. A PCI-6711E (National Instruments) board was used to synchronize the waveform acquisition, data collection and stimulation delivery.

Background-subtracted cyclic voltammograms were obtained by digitally subtracting voltammograms collected during stimulation from those collected during baseline recording. Electrical stimulation was triggered after a synchronization signal was sent to the external input of a Master-8 pulse generator (A.M.P.I. Jerusalem, Israel). Voltages generated by the Master-8 were converted to constant currents via a stimulus isolation unit (AM-2200, AM-Systems, Carlsborg, WA, USA). Electrical stimulation was delivered 5 s after the start of each recording, and each was delivered in the 91.5 ms inter-waveform interval so that it did not interfere with the voltammetry scans.

Once the carbon fiber electrode was in place, electrical stimulation (40 cathodal rectangular, constant-current pulses, 0.1 ms in duration) was delivered at a frequency of 98 Hz at the current intensity used during the behavioral tests or at an initial current of 400 μA.) through the DR electrode while DA transients were monitored; if a DA signal was not observed, the working electrode was lowered by 0.1 mm, and the electrical stimulation was repeated. This sequence was reiterated until stimulation-induced DA transients were detected. For naïve rats, the current intensity was adjusted so that the magnitude of DA signal measured across the different stimulation parameters was similar to that of the behaviorally trained rats. The DA signal was recorded in response to a descending set of pulse parameters similar to the ones used during the behavioral test. Each set of stimulation parameters was repeated three times with a 60-s inter-stimulation interval. This inter-stimulation interval produces a stable amplitude of DA transients (Cossette et al., 2016). Following this initial sweep, stimulation was delivered using three sets of parameters, corresponding to those that had produced maximal, half-maximal, and minimal responding in the behavioral test. The first sweep was used as the baseline, and then the vehicle was injected into the VM, followed by PPPA; The DA release was monitored across the three sets of stimulation parameters. In some rats, apomorphine or its vehicle was injected systemically (s.c.) after PPPA, and DA release was monitored again. At the end of the FSCV recordings, electrodes were post-calibrated by placing the electrode into a flow injection system (Upchurch Scientific, Oak Harbor, WA, USA), in which known concentrations of DA 100, 200, and 500 nM dissolved in artificial cerebrospinal fluid (aCSF: 145 mM Na^+^, 2.7 mM K^+^, 1.22 mM Ca^2+^, 1.0 mM Mg^2+^, 150 mMCl^−^, 0.2 mM ascorbate, 2 mM Na_2_HPO_4_, pH 7.4 ± 0.05) were used to related current values to concentration values. The average concentration of DA transients observed in the NacS after electrical stimulation of the DR at maximum frequency was 151.38 (SEM = 19.58) nM a value in the range of previously reported studies (Cheer et al., 2007; Park et al., 2010; Oleson et al., 2012; Saddoris et al., 2015).

### Histology

After the completion of the experiment the location of the cannulae, stimulating and recording electrodes were obtained via deposit of iron ions after 1 mA of anodal current was delivered for 60 s. Animals were deeply anesthetized with urethane (2 g/kg, i.p.) and the current was delivered through the stimulating electrode, through the injection cannulae that were inserted into the guides, or through a stimulating electrode that was lowered to the site at which the voltammetric recordings were obtained. The animals were then perfused intracardially with 0.9% sodium chloride, followed by a formalin-Prussian Blue solution (10% formalin, 3% potassium ferricyanide, 3% potassium ferrocyanide and 0.5% trichloroacetic acid) that forms a blue reaction product with the iron particles. Brains were removed and fixed with 10% formalin solution for at least 7 days. Coronal sections of 40-μm thickness was cut with a cryostat to confirm placements of the FSCV electrodes in the NAcS, the stimulating electrode in the DR and cannulae in the VM.

### Data Analysis

Matlab (Natick, MA, USA) was used to fit curves to the nose-poke and DA-release data. The phasic DA release was normalized to the maximum current obtained during baseline recordings. When *standardized*, nose-poke responses were normalized to the maximum number of responses. Comparisons between effectiveness indices for the behavioral and neurochemical data were made with paired or unpaired Student’s *t*-tests or one-way repeated-measures ANOVAs, followed by Tukey’s honestly significant difference *post hoc* tests. If the sphericity assumption was violated, the Greenhouse and Geisser correction was used. The correlation between the average change in M50 and the average change in the DA phasic release following PPPA injection was computed. Initial voltammetric data analysis was performed using LabVIEW written software and was low-pass filtered at 2 KHz. DA was chemically identified by its characteristic background-subtracted cyclic voltammogram; oxidation peak occurs at ~0.65 V and the reduction peak at ~−0.2 V, and principal component regression (PCR) was used as previously described to extract the DA component from the raw voltammetric data (Heien et al., 2005). The DA signal used for analysis was time-locked to the electrical stimulation trains. The quality of the signature was comparable at all pulse frequencies. All analyses were performed and graphics were prepared in Origin v9 (Northampton, MA, USA).

## Results

### Histology

**Figure 2:**
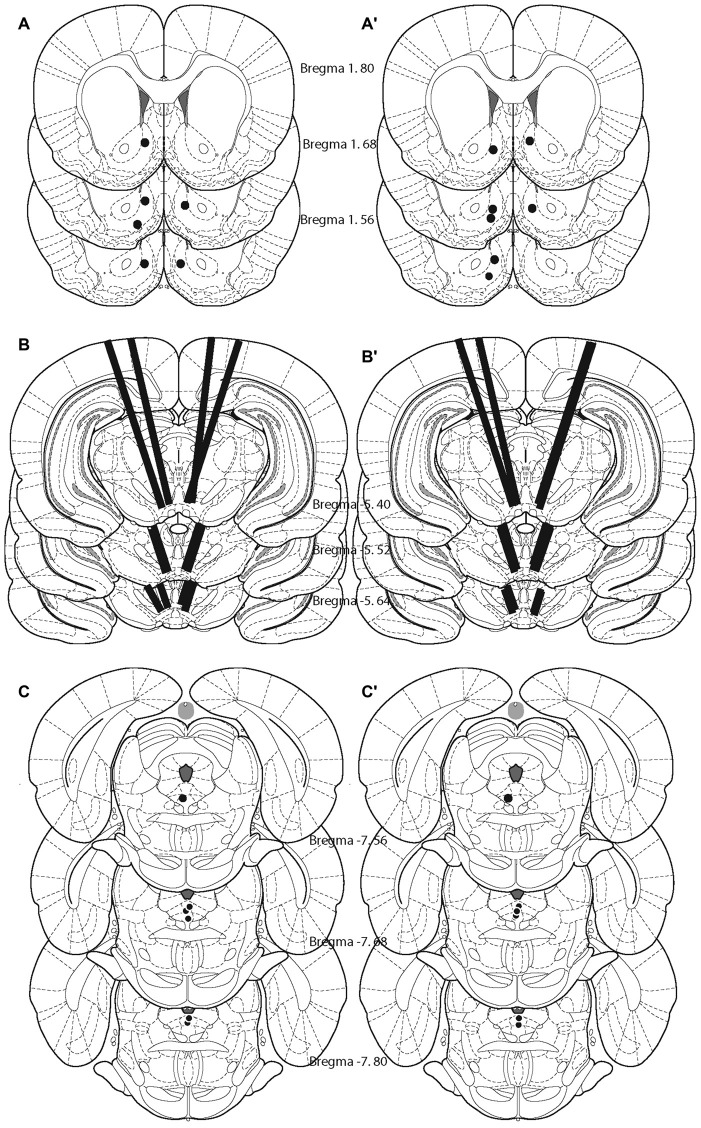
**Location of the tips of the carbon fibers (fast-scan cyclic voltammetry, FSCV; A,A′), injection sites (B,B′), and stimulating electrodes (C,C′) for each animal included in the study.** Left panel shows the animals that were behaviorally trained. Right panel shows naïve animals.

Histological analysis revealed that the tips of the FSCV recording electrodes were within the shell of the nucleus accumbens (Figures 2A,A′). The injection sites were located within the ventral part of the VM (Figures 2B,B′), a region that contains neurons activated by rewarding electrical stimulation (Wise and Rompre, 1989; Marcangione and Rompré, 2008). Finally, stimulation sites were located within the postero-medial mesencephalon, within the ventral central gray, between the anterior-posterior regions (Figures 2C,C′).

### Sweep Dopamine Transients

**Figure 3:**
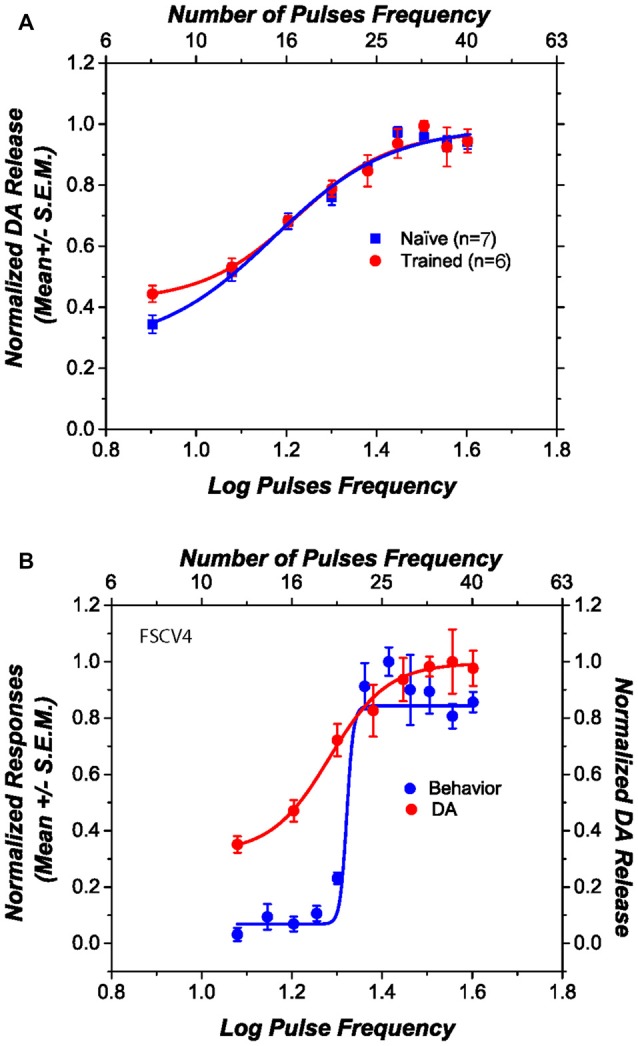
**Dopamine (DA) release induced by dorsal raphe (DR) stimulation, as a function of pulse frequency. (A)** The DA release profile obtained from behaviorally trained and naïve subjects is very similar. **(B)** The relation between self-stimulation performance and stimulation-induced DA release in one representative subject. Although the slopes of the two curves differ, their midpoints fall at similar positions along the pulse-frequency axis.

Figure 3A shows the obtained DA phasic transiens across the different stimulation parameters for those rats that received previous behavioral training (circles) and those that were naïve (squares). The DR electrical stimulation induced DA transients in the NAcS and the magnitude of the transients increased systematically as a function of the pulse frequency. Normalized phasic DA transients overlap, and there is no statistical difference in the steepness of the curves [*t*_(11)_ = 1.09; *p* > 0.05], the pulse frequency that produced half-maximal release [*t*_(11)_ = 1.66; *p* > 0.05], the lower asymptote [*t*_(11)_ = 0.57; *p* > 0.05], or the upper asymptote [*t*_(11)_ = 0.89; *p* > 0.05] between trained and naïve subjects. Figure 3B shows, in one subject, how self-stimulation performance and stimulation-induced DA transients vary as a function of pulse frequency. Although the slopes, in all the behaviorally tested subjects, between fitted curves relating stimulation frequency to the behavior and DA release differ [*t*_(5)_ = 3.79; *p* < 0.05]; their M50 values do not [*t*_(5)_ = 1.78; *p* > 0.05].

### PPPA Enhanced Brain Stimulation Reward, Yet it Decreased Dopamine Transients

**Figure 4:**
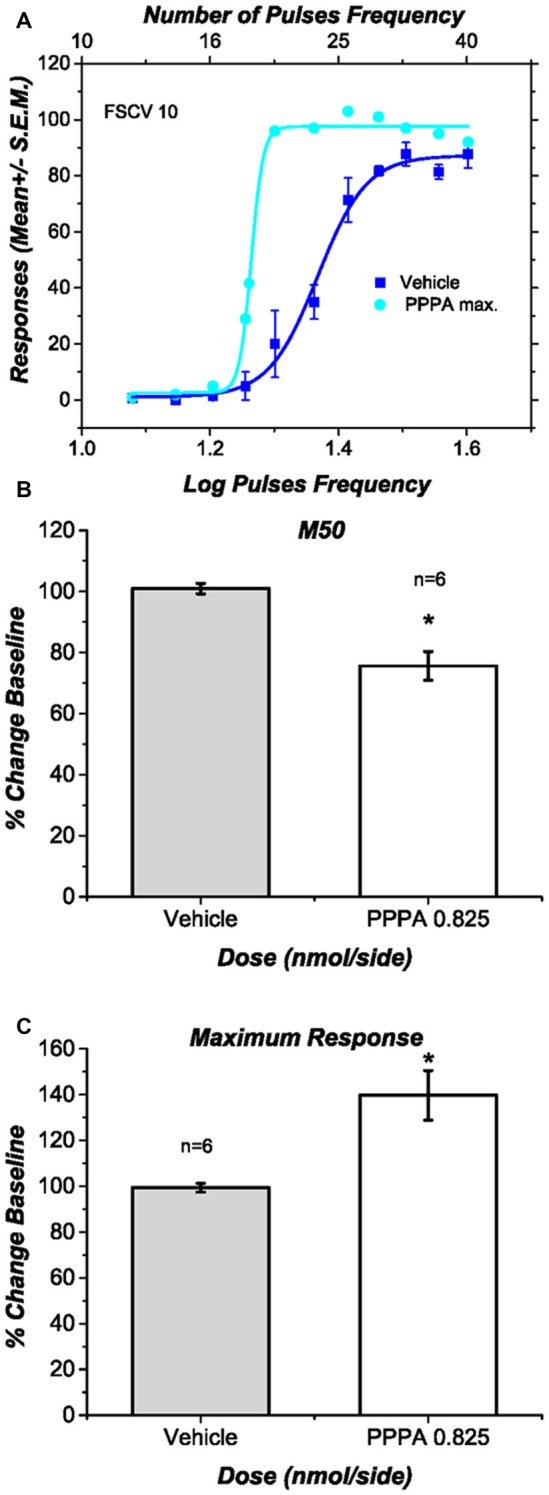
**Effects of intra ventral midbrain (VM) (2R,4S)-4-(3-Phosphopropyl)-2-piperidinecarboxylic acid (PPPA) injection on behavior. (A)** In one representative subject intra VM injection of PPPA shifted the response-rate vs. pulse-frequency curve leftward and increased its upper asymptote. **(B)** The bar graph shows a significant reduction in M50 value in all the behaviorally trained subjects in which DA release was successfully measure. The reduction in M50 value suggests an increase in the effectiveness of the stimulation to elicit nose-poke behavior. **(C)** The bar graph shows a significant increase in the maximum response rate elicited by intra VM injection of PPPA.

Figure 4A shows for a representative subject the behavioral effects of intra-VM injections of PPPA (0.825 nmol/0.5 μl/side). This drug produced a leftward and upward shift of the curve that relates the nose-poke rate as a function of pulse frequency: less stimulation was necessary to obtain a given level of performance and more responses were emitted when contrasted against the vehicle, at some stimulation frequencies. Figures 4B,C show respectively the average changes in M50 values and in maximal response expressed as a percentage of baseline, for all the behaviorally trained subjects in which DA transients were recorded. In contrast to the vehicle, PPPA produced a significant 25.3% (SEM = 6.3) reduction in M50 value [*t*_(5)_ = 3.43; *p* < 0.05] and a significant 39.7% (SEM = 11.7) increase in nose-poke responses [*t*_(5)_ = 3.27; *p* < 0.05]. The observed potentiation of DR reward is very similar to what has been previously observed and described (Bergeron and Rompré, 2013; Ducrot et al., 2013; Hernandez et al., 2015).

**Figure 5:**
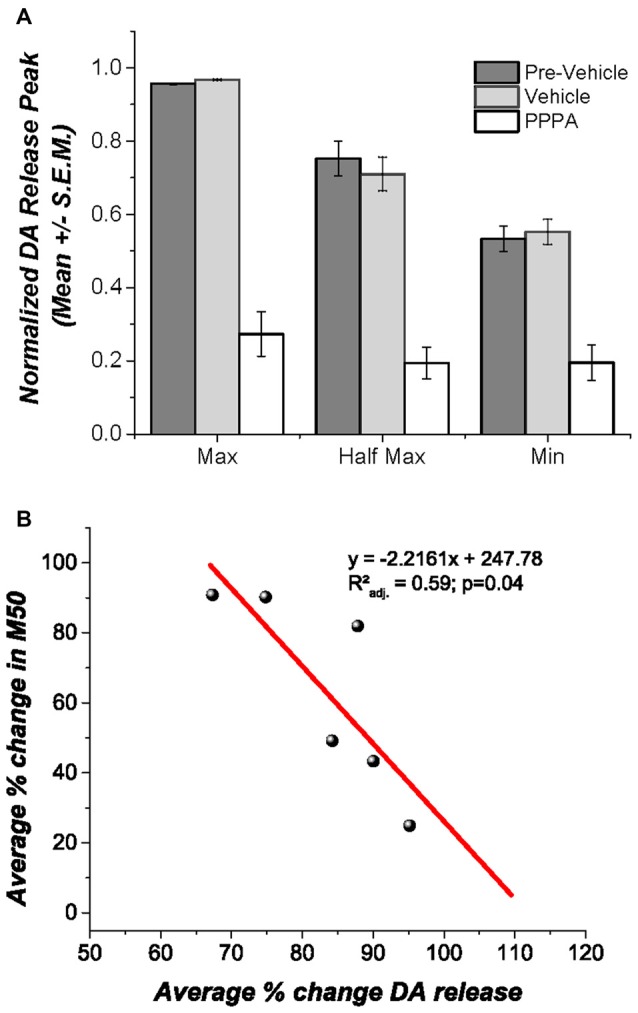
**Effects of intra VM PPPA injection on DA release. (A)** Under urethane anesthesia intra VM PPPA injection decreased stimulation-elicited DA release in comparison to measures obtained before and after vehicle injection; this effect was seen at all three frequencies tested. **(B)** A negative correlation between the drug-induced decrease in stimulation-evoked DA release and the change in M50 was observed. In the behaviorally trained animals, the enhancement in reward effectiveness, produced by VM injection of PPPA covariates with the reduction in DA release; so that the larger the enhancement the greater the DA release reduction.

Under urethane anesthesia (Figure 5A) VM vehicle microinjection produced no discernable change in DA transients evoked by DR stimulation, whereas PPPA injection produced a significant decrease in the magnitude of the DA transients at all tested frequencies Maximum [*F*_(1.002,5.02)_ = 47.94; *p* < 0.05]; M50[*F*_(1.09,5.46)_ = 12.48; *p* = <0.05]; Minimum [*F*_(1.41,7.07)_ = 15.65; *p* < 0.05]. It is noteworthy that the decrease in DA is negatively correlated with the magnitude of reward enhancement (*r*_xy_ = −0.821); 59% of the variance observed in the magnitude attenuation of DA transients can be explained by the observed magnitude of reward enhancement (*r*^2^_adj._ = 0.593; *p* < 0.05, Figure 5B).

**Figure 6:**
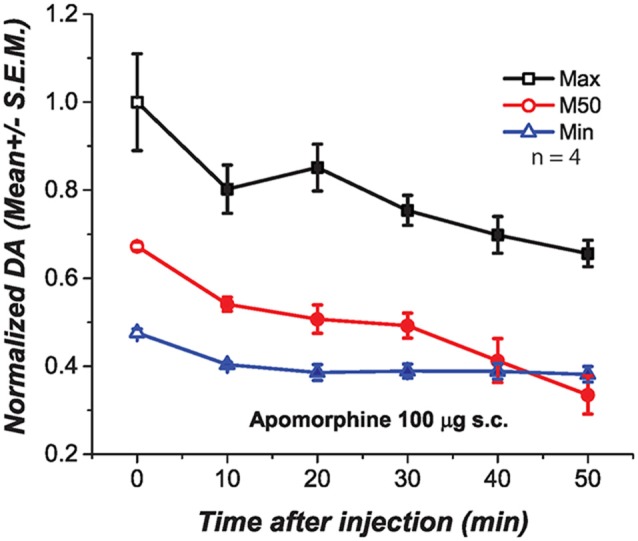
**Subcutaneous injection of apomorphine (100 μg) produced a significant and long lasting reduction of DA oxidation at the three frequencies tested.** Filled symbols represent a significant reduction of DA oxidation when contrasted against the pre-injection values, time 0.

We injected apomorphine to test whether the negative correlation between the drug-induced changes in M50 and DA transient magnitude resulted from depolarization inactivation (DI) in DA neurons due to the strong excitatory drive produced by the DR stimulation. At low doses, apomorphine is known to reduce DA firing by stimulating DA autoreceptors. This action hyperpolarizes DA neurons and increases input resistance, restoring DA firing and excitability when neurons are in a state of DI (Grace and Bunney, 1985). We injected a dose of apomorphine (100 μg s.c., Figure 6) that when injected alone produced a long-lasting and significant reduction of DA transients at the three frequencies tested Maximum [*F*_(1.65,4.96)_ = 9.91; *p* < 0.05]; M50[*F*_(1.89,5.69)_ = 17.87; *p* < 0.05]; Minimum [*F*_(1.41,7.07)_ = 8.04; *p* = <0.05].

**Figure 7:**
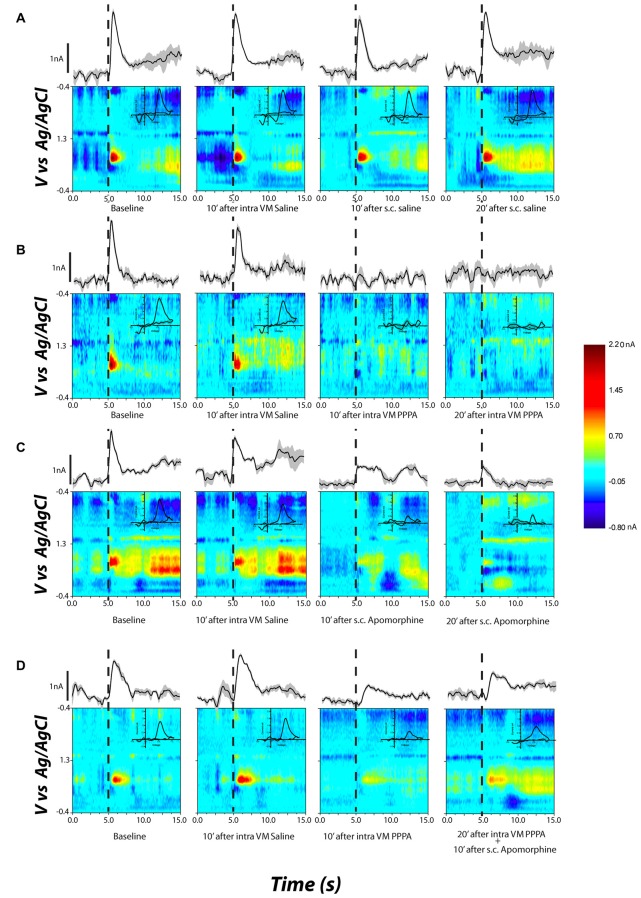
**Examples of DA release in the nucleus accumbens shell (NAcS) in response to electrical stimulation of the DR at M50 parameters.** The false-color plots show redox currents as function of applied voltage and time. Current at the peak oxidation potential of DA is shown above the false-color plot as function of time. The insets show the cyclic voltammogram. Dashed lines denote the onset of the electrical stimulation train. **(A)** DA release for M50 stimulation at baseline, 10 min after intra-VM saline; 10 and 20 min after s.c. saline. The DA peak is quite stable over time and across conditions. **(B)** Intra-VM injection of PPPA alone produces a significant decrease in DA release. **(C)** A similar decrease in DA phasic release is observed after s.c. injection of apomorphine. **(D)** Administration of apomorphine 20 min after intra-VM injection of PPPA partially restored the magnitude of DA transient.

Figure 7 documents, for different selected subjects, the effects of apomorphine on stimulation-induced DA transients. The false-color plots represent background-subtracted redox currents as a function of voltage and time. Above each false-color plot is the time course of the DA-oxidation current at the potential corresponding to peak oxidation (the ordinal value corresponding to the center of the red blob in the leftmost false-color plot). DA transient concentration is directly proportional to this oxidation current. Superimposed on the false-color plots, in the upper right quadrant, is a background-subtracted voltammogram. The form of the voltammograms matches the voltammetric signature of DA; oxidation peak occurs at ~0.65 V and the reduction peak at −0.2 V. The time-course plots and voltammograms are horizontal and vertical sections, respectively, passing through the peak DA-oxidation current in the false-color plot. The dashed line denotes the onset of the electrical stimulation train.

Figure 7A (top row) shows that systemic or intra-VM injection of saline had a negligible effect on the stimulation-induced DA transient. The rightmost two panels in Figures 7B,C demonstrate that both intra-VM PPPA (Figure 7B) and systemic apomorphine (Figure 7C) decreased the stimulation-induced DA transient. Systemic administration of apomorphine 10 min following intra-VM injection of PPPA partially restored the DA transient (Figure 7D, 4th column).

**Figure 8:**
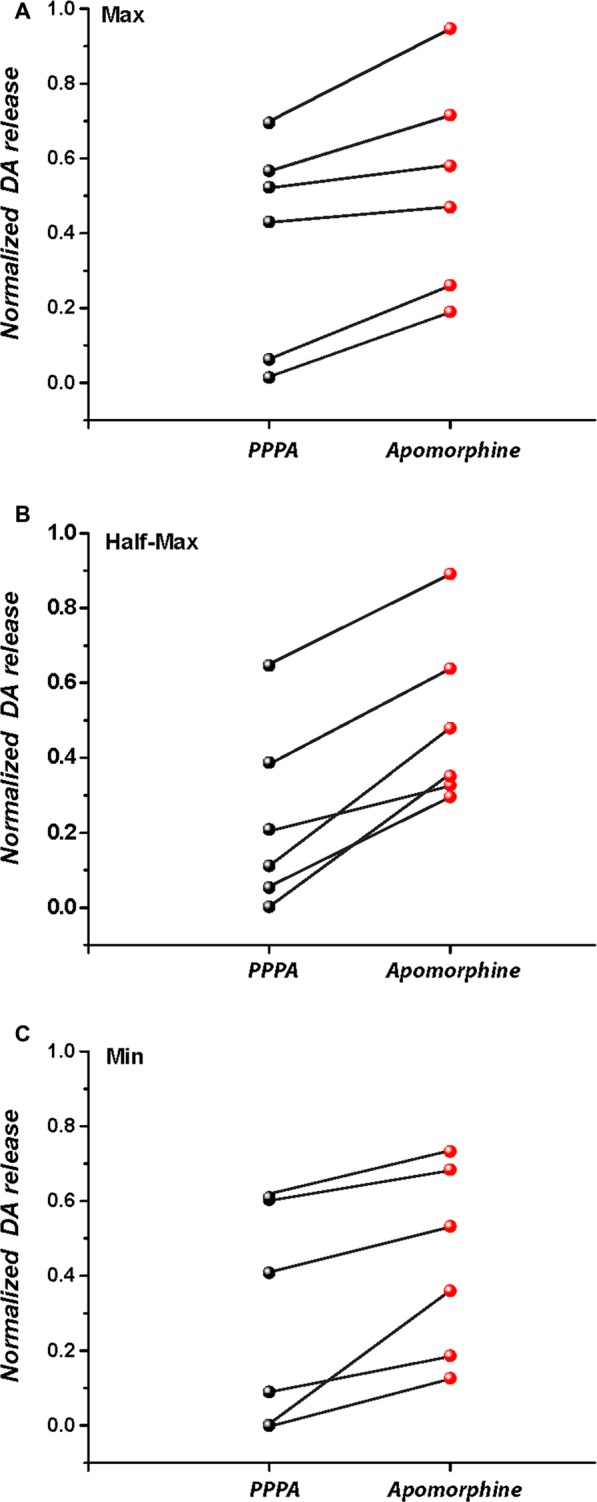
**Subcutaneous injection of apomorphine 20 min after intra-VM injection of PPPA partially restores stimulation-induced DA release at the maximal stimulation frequency (A), M50 stimulation frequency (B), and at the lowest stimulation frequency (C)**.

Figure 8 quantifies the partial restoration of the DA transients by systemic administration of apomorphine (as illustrated by the difference between the peak DA oxidation currents in the bottom right panel of Figure 7 and the panel to its immediate left). The increase observed at each frequency was significant when contrasted against the last transient recorded after PPPA injection (*t*_Max(5)_ = 4.34, *p* = <0.05; *t*_M50(5)_ = 7.17, *p* < 0.05; *t*_Min(5)_ = 3.58, *p* < 0.05).

## Discussion

DA plays a critical role in reward and reward-seeking. Direct optical stimulation of DA neurons produces a conditioned place-preference (Tsai et al., 2009) and rodents will perform an operant response to optically activate VM DA neurons (Witten et al., 2011; Kim et al., 2012). Rewarding electrical stimulation of different brain areas increases DA levels in the Nac and elicits DA phasic release (Hernandez et al., 2006; Owesson-White et al., 2008; Hernandez and Shizgal, 2009; Cossette et al., 2016). Also, drugs that boost DA release enhance brain stimulation reward (BSR); whereas the opposite behavioral effect is obtained with drugs that decrease DA availability (Wise and Rompre, 1989). Our results show that electrical stimulation of the DR, at frequencies that elicit self-stimulation behavior, produces DA phasic release in the NacS. The magnitude of DA transients increase as a function of pulse frequency in an orderly manner and eventually levels off, as it has been observed previously (Fiorino et al., 1993; Yavich and Tanila, 2007). In our anesthetized preparation, DA release correlates with the behavioral performance observed during self-stimulation, which suggests that information about the reward intensity is reflected in the DA phasic release, at least over the range of stimulation parameters tested in the present study. To evaluate this hypothesis rigorously, it will be necessary to determine whether equiprefered stimulation parameters would produce similar phasic DA output (Moisan and Rompre, 1998).

In freely moving animals, preferential blockade of VM NMDA GluN2A receptors with the PPPA enhances the reward-seeking produced by electrical stimulation of the DR (Bergeron and Rompré, 2013; Ducrot et al., 2013; Hernandez et al., 2015). This enhancement implicates GluN2A receptors in inhibition of VM DA neurons. Blockade of these NMDA receptors will lead to a disinhibition of DA neurons, enhanced phasic firing and DA release in terminal areas. Unexpectedly, electrically induced DA transients were lost when rewarding stimulation was combined with VM microinjection of PPPA. One possible explanation for this unexpected result is the product of an increase in net excitation that drives the DA neurons into a state of DI (White and Wang, 1983; Grace and Bunney, 1986). To test this hypothesis, we determined whether apomorphine can restore electrically induced DA transients following microinjection of PPPA. An *in vitro* electrophysiological study has shown that under normal conditions, apomorphine hyperpolarizes DA neurons (Grace and Bunney, 1985). However, in a state of DI, apomorphine-induced hyperpolarization restores the responsiveness of DA neurons to excitatory input (Grace and Bunney, 1986) by allowing the slow sodium gates to reopen. Consistent with this hypothesis, apomorphine partially restored the magnitude of electrically induced DA transients in animals that had received a prior VM microinjection of PPPA. We speculate that DA release was not totally restored because apomorphine produces multiple effects, some of which have opposing influences on the excitability of DA neurons. In addition to hyperpolarization due to stimulation of DA autoreceptors, apomorphine activates nerve-terminal autoreceptors, an effect that reduces extracellular DA release (Grace and Bunney, 1985). Accordingly, we found that apomorphine reduced the magnitude of electrically induced DA transients following VM microinjection of the vehicle.

The occurrence of DI in our preparation is most likely the product of coordinated disinhibition, via blockade of NMDAR, and glutamatergic excitation, via activation of AMPA receptors (Ducrot et al., 2013; Qi et al., 2014). Similar synergistic effects leading to DI had been previously reported with the DA antagonist, pimozide, and the opiate agonist, morphine (Rompre and Wise, 1989; Henry et al., 1992). A VM microinjection of morphine that enhanced BSR in naïve animals produced a complete cessation of responding in animals previously injected with pimozide. In this latter condition, operant responding was reinstated by VM microinjection of a dose of the GABA agonist, muscimol, that inhibited reward under a control condition (no other drug treatment).

The present findings not only support the hypothesis that PPPA and reward synergize to enhance DA excitation, but they also suggest that different NMDAR receptor subtypes are involved in modulation of mesoaccumbens DA impulse flow. NMDARs are heterodimers composed of two GluN1 subunits with GluN2 and/or GluN3 subunits. Previous pharmacological and SiRNA data suggest that PPPA-sensitive NMDARs are most likely located on VM afferents to DA neurons, are composed of GluN2A subunits, and are devoid of GluN2B (Bergeron and Rompré, 2013; Hernandez et al., 2015). Although previous results show that the DR reward signal is transmitted to VM neurons through AMPA receptors, a role for NMDAR cannot be excluded. NMDAR activation is essential for induction of DA burst firing (Zweifel et al., 2009) and a reduction in VM GluN1, the subunit common to all NMDARs, produces a significant attenuation of DR reward (Hernandez et al., 2015); it is thus most likely that the NMDAR involved induction of DA burst firing is composed of different subunits than the NMDAR that controls the inhibitory drive.

The present results show that among its many roles, glutamate mediates a strong inhibitory drive on DA neurons and that DA-related reward signals can be strongly enhanced by reducing this inhibitory drive through blockade of VM NMDARs. This provides additional evidence that glutamate modulates DA neural activity in multiple ways and thus plays a key, albeit complex, role in reward signaling.

## Author Contributions

P-PR and GH designed the study. GH and M-PC carried out the experiments, GH analyzed the data. P-PR, GH, M-PC and PS contributed to interpretation of the results and writing of the article.

## Funding and Disclosure

This article was supported by Natural Sciences and Engineering Research Council of Canada (NSERC) grants to P-PR (#119057, RGPIN-2015-05018) and to PS (RGPIN-308-11); NSERC postdoctoral Fellowship to GH, a grant from the “Fonds de recherche du Québec—Santé” to the “Groupe de Recherche en Neurobiologie Comportementale”/Center for Studies in Behavioral Neurobiology, and support to PS from the Concordia University Research Chairs program.

## Conflict of Interest Statement

The authors declare that the research was conducted in the absence of any commercial or financial relationships that could be construed as a potential conflict of interest.
